# Human perinatal immunity in physiological conditions and during infection

**DOI:** 10.1186/s40348-017-0070-1

**Published:** 2017-04-21

**Authors:** Gijs T. J. van Well, Leonie A. Daalderop, Tim Wolfs, Boris W. Kramer

**Affiliations:** 1 Department of Pediatrics, Division of Infectious Diseases and Immunology, Maastricht University Medical Center (Maastricht UMC+), Maastricht, The Netherlands; 2 Department of Pediatrics, Laboratory of Pediatrics, Maastricht University Medical Center (Maastricht UMC+), Maastricht, The Netherlands; 3 Department of Pediatrics, Division of Neonatology, Maastricht University Medical Center (Maastricht UMC+), Maastricht, The Netherlands; 4 School for Nutrition and Metabolism (NUTRIM), Maastricht University Medical Center (Maastricht UMC+), Maastricht, The Netherlands; 5 School for Developmental Biology and Oncology (GROW), Maastricht University Medical Center (Maastricht UMC+), Maastricht, The Netherlands

## Abstract

The intrauterine environment was long considered sterile. However, several infectious threats are already present during fetal life. This review focuses on the postnatal immunological consequences of prenatal exposure to microorganisms and related inflammatory stimuli. Both the innate and adaptive immune systems of the fetus and neonate are immature, which makes them highly susceptible to infections. There is good evidence that prenatal infections are a primary cause of preterm births. Additionally, the association between antenatal inflammation and adverse neonatal outcomes has been well established. The lung, gastrointestinal tract, and skin are exposed to amniotic fluid during pregnancy and are probable targets of infection and subsequent inflammation during pregnancy. We found a large number of studies focusing on prenatal infection and the host response. Intrauterine infection and fetal immune responses are well studied, and we describe clinical data on cellular, cytokine, and humoral responses to different microbial challenges. The link to postnatal immunological effects including immune paralysis and/or excessive immune activation, however, turned out to be much more complicated. We found studies relating prenatal infectious or inflammatory hits to well-known neonatal diseases such as respiratory distress syndrome, bronchopulmonary dysplasia, and necrotizing enterocolitis. Despite these data, a direct link between prenatal hits and postnatal immunological outcome could not be undisputedly established. We did however identify several unresolved topics and propose questions for further research.

## Introduction

Healthy living has its origin before birth. Although the intrauterine setting is considered to be a safe environment for the unborn, several threats are already present. One of them is exposure to microorganisms and subsequent inflammatory responses in both maternal (chorionic) and fetal (amniotic) tissues [1]. Antenatal inflammatory responses to infectious stimuli may have positive or negative effects on the fetus and potentially influence its immune responses later in life. The immune system of both the fetus and the (preterm) neonate itself has limited capacity because of gestational immaturity and needs stimulation to develop [2]. Fetuses and neonates therefore rely to a significant extent on their innate immune system [3, 4]. This might enhance neonatal vulnerability to infection but may also protect against collateral inflammatory damage. Although several infectious challenges are present, even prenatally, and immune responses are downregulated, not all fetuses and neonates will develop serious infections. Intrauterine infections may induce premature delivery; prematurity raises the risk for serious neonatal infections, but it is unknown whether this is independently correlated with immature immune responses.

## Review

What can we learn from the nature of immune responses in preterm and term neonates with and without infections? In other words, do prenatal microbial challenges affect postnatal immunity and if so, how?

This review focuses on postnatal immunological consequences of prenatal exposure to microorganisms and other inflammatory stimuli. We will also summarize what is known about the microbial triggers and inflammatory consequences of clinically well-known neonatal immune-mediated diseases, such as respiratory distress syndrome (RDS), bronchopulmonary dysplasia (BPD), and necrotizing enterocolitis (NEC).

## Methods

The electronic databases MEDLINE (PubMed) and Google Scholar were searched. Articles related to fetal and neonatal immunity and infection, as well as articles related to postnatal immunological consequences of prenatal infection/inflammation, were acquired. We did not intend to perform a systematic review and focused on identification of unresolved topics and questions for further research.

## The fetal immune response

Fetal immune responses rely largely on innate immunity because the adaptive immune potential is still not fully matured at that stage of life [3–5].

### The developing immune cells and tissues

The development of the human immune system starts with the production of hematopoietic stem cells (HSCs) during embryogenesis, which have the ability to differentiate into myeloid and lymphoid cells [6]. At weeks 4–6 of gestation, HSCs migrate to the liver, which becomes the main site of hematopoiesis until week 22 of gestation. HSCs colonize the thymus at week 8 of gestation and bone marrow between 70 and 80 days of gestation [5, 7]. During the third trimester, hepatic hematopoiesis decreases and terminates shortly after birth [2]. The development of secondary lymphoid tissues (e.g., spleen, lymph nodes, and Peyer’s patches) starts at week 8 of gestation [8].

### Innate immunity

Embryonic macrophages are the first immune cells found in the yolk sac at weeks 3–4 of gestation [9, 10]. The majority of macrophages are still negative for the important major histocompatibility complex (MHC)-II. MHC-II-positive macrophages start to appear in the liver around weeks 7–8 of gestation, in the lymph nodes between weeks 11 and 13 of gestation, and in the developing thymic medulla around week 16 of gestation [11].

Two months after conception, monocytes are present in high quantities in the circulation as a result of established hematopoiesis in the fetal liver. Their amount, phagocytic function, and antigen presenting capacity increase with gestational age (GA) [5, 10].

In contrast to the high levels of circulating monocytes, neutrophil levels are low before 31 weeks of GA. After 31 weeks, they increase substantially [12].

Fetal blood samples collected between 21 and 32 weeks of GA showed that the functionality of neutrophils is impaired during fetal life. Fetal neutrophils express lower amounts of adhesion molecules compared to neonatal and adult neutrophils, which reduces their ability to adhere and extravasate from the bloodstream. As a consequence, their chemotactic ability is limited [13, 14]. Their ability to migrate gradually increases after 32 weeks of GA, and in full-term neonates, migration ability reaches adult levels.

Dendritic cells (DCs) act as a link between innate and adaptive immunity and originate from both myeloid and lymphoid progenitor cells [15].

Also of significant importance in the early phases of life is the natural killer (NK) cell. Although derived from bone marrow lymphoid progenitor cells, NK cells are classified between innate and adaptive immunity. They are detectable from 6 weeks of GA onwards in the embryonic liver [16]. The amount of NK cells correlates with GA. Levels reach a maximum at birth [5]. The cytolytic function of fetal NK cells is considerably lower compared to that of adults. Even at term, the cytolytic capacity is only 50–80% of adult levels [17].

### Adaptive immunity

The thymus is the T cell-producing organ and starts to develop during week 6 of gestation. Progenitor T cells start to colonize the fetal thymus around week 8 of gestation [18]. At week 15 of gestation, fetal thymocytes express a complete set of TCRs [18]. The amount of T cells starts to increase from 19 weeks of gestation and peaks at about 6 to 7 months postnatally [18].

B cells are derived from HSCs in the bone marrow and fetal liver. Pre-B cells are detectable in the fetal liver from week 7 of gestation and in the bone marrow around week 12 [19–21]. At the time B cells express immunoglobulin M (IgM) on their surface, they start to migrate from the bone marrow to the peripheral circulation. IgM-positive B cells are present in the peripheral circulation by week 12 of gestation. Between weeks 10 and 12 of gestation, different immunoglobulin isotypes start to appear in the peripheral circulation: B cells with surface immunoglobulin D (IgD), surface immunoglobulin G (IgG), and surface immunoglobulin A (IgA). During fetal life, the number of B cells increases to adult levels by week 22 of gestation. The amount of plasma cells is low until weeks 18–20 of gestation [19]. The fetus is already able to mount an antigen-specific antibody response; however, this response is at a lower intensity compared to that of adults. Neonatal antibody responses depend on the type of antigen, with better responses to proteins compared to polysaccharides. Antibody responses to polysaccharide antigens only reach mature levels after 2 years of age in childhood [22, 23].

The adaptive immune system and T cell immunity in particular is still immature in the fetus [3]. Adaptive responses in general are downregulated in the fetal stage of development. The biological function of this phenomenon is related to the sophisticated immune balance between the mother and the fetus. Mechanisms that control downregulation of adaptive immunity in mother and fetus include local immunosuppression, lack of DC migration to local lymph nodes, and presence of intrauterine Tregs during pregnancy [24, 25]. The delicate process of fetal development should evidently not be disturbed by potentially harmful inflammatory responses. To avoid inflammation, fetal cells such as macrophages are hyporesponsive, and soluble inflammatory mediators are scarce. Additionally, the fetal adaptive immune response is characterized by suppressed Th1 responses and upregulated Th2 responses. This shift from Th1 to Th2 is caused by the production of mediators through the placenta, such as the anti-inflammatory cytokine interleukin (IL)-10, prostaglandin E2, and progesterone [4].

A typical cytokine profile characterizing fetal immune responses consists of low levels of type I IFNs, such as IFN-α and IFN-γ; IL-12 and higher levels of innate inflammatory cytokines IL-1β, IL-6, IL-23; and much higher anti-inflammatory IL-10 levels [4].

## Prenatal infection and immunity

The intrauterine environment was long considered to be sterile. This classical dogma has been questioned over the past decade, and there is evidence that gut microbiota colonization already starts in utero [26, 27]. Microbes have been found in placental tissue, umbilical cord blood, fetal membranes, amniotic fluid, and meconium. It remains unclear how this colonization occurs. It is possible that microbes originate from the vagina or maternal digestive tract. A study by Aagaard et al. [28] showed that the placental microbiome is most akin to the human oral microbiome. This study makes it plausible that microbes may also originate from the oral cavity of the mother.

Intrauterine infections are a leading cause of preterm birth [1]. However, it is also well established that the presence of bacteria in the amniotic fluid does not always result in preterm delivery, nor does it always induce chorioamnionitis (CA) [29]. CA occurs most often as a result of ascending bacteria from the vagina and cervix [1, 30]. CA manifests clinically as maternal fever, maternal and fetal tachycardia, uterine tenderness, abnormal vaginal discharge, and leukocytosis. The majority of fetuses exposed to CA develop a systemic inflammatory response known as the *fetal inflammatory response syndrome* (FIRS) [31]. This often causes preterm rupture of the membranes, and therefore, CA is strongly associated with premature birth. CA is histologically characterized by inflammation and necrosis throughout the chorion and amnion [30]. Successive histologic stages of the fetal immune response are proposed by the Society for Pediatric Pathology working group on amniotic fluid infections (displayed in Table 1) [32, 33].

**Table 1:** Histological stages of the fetal immune response towards chorioamnionitis [32, 33]

Histological stage	Characteristics
1	*Stage 1* is defined by neutrophils in the chorionic vessels (*chorionic vasculitis*) and/or umbilical vein (*umbilical phlebitis*).
2	*Stage 2* is reached when neutrophils enter the wall of the umbilical artery (*umbilical arteritis*), with or without minor degrees of extravasation into Wharton’s jelly.
3	*Stage 3* is heralded by organization of neutrophils in Wharton’s jelly into arc-like bands surrounding one or more umbilical vessels (concentric umbilical perivasculitis or necrotizing funisitis).

Gomez et al. [34] showed that fetuses with severe neonatal morbidity had a lower mean GA and a higher proportion of CA. Furthermore, these fetuses had significantly higher levels of IL-6 in amniotic fluid and fetal plasma [34].

### Preterm birth

Preterm birth may result from either spontaneous developments or medically indicated interventions. Although a large proportion of preterm births are labeled idiopathic, i.e., without known cause, there is good evidence that infections are a primary cause of a substantial proportion of preterm births [35] since (a) the amniotic fluid of women with preterm labor has higher rates of microbial colonization and levels of inflammatory cytokines than that of women not in labor and women in labor; (b) extrauterine maternal infections such as pyelonephritis, pneumonia, and periodontal disease have been associated with premature parturition; (c) subclinical intrauterine infections are associated with preterm labor and delivery; and (d) patients with intra-amniotic infection or intrauterine inflammation are at risk for subsequent preterm delivery [35]. Additionally, pro-inflammatory cytokine production (IL-1β, IL-6, tumor necrosis factor (TNF)-α) in response to intrauterine infection and inflammation is more likely to cause preterm labor than anti-inflammatory cytokine production (IL-10) [35]. Romero et al. [36] showed evidence for the contribution of IL-1β to preterm birth: (1) IL-1β is produced by human decidua in response to bacterial products; (2) IL-1β can stimulate prostaglandin production of human amnion and decidua; (3) IL-1β concentration and bioactivity were increased in the amniotic fluid of women with preterm labor and infection; and (4) IL-1β could stimulate myometrial contractions [36]. They also showed evidence for a supporting role of TNF-α in preterm birth [36]. This was based on the following observations: (1) TNF-α stimulates prostaglandin production by amnion, decidua, and myometrium; (2) human decidua can produce TNF-α in response to bacterial products; (3) amniotic fluid TNF-α bioactivity and immunoreactive concentrations are elevated in women with preterm labor and intra-amniotic infection; (4) in women with preterm premature rupture of membranes (PPROM) and intra-amniotic infection, TNF-α concentrations are higher in the presence of labor; (5) TNF-α can stimulate the production of matrix metalloproteinases (MMPs), which may play a role in membrane rupture and cervical ripening; and (6) TNF-α application in the cervix induces changes that resemble cervical ripening [36].

There is growing evidence that normal spontaneous labor at term age is an inflammatory process on itself [37–40], but the extent to which the mechanisms of normal and preterm labor (abnormal) overlap remains largely unknown [35].

## The neonatal immune response

Neonates are highly susceptible to infectious agents. Limited exposure to antigens in utero and the lack of preexisting immunological memory contribute to this sensitivity [3]. Additionally, pro-inflammatory responses are suppressed in neonates [41, 42]. Therefore, neonates rely heavily on their innate immune system in fighting infections [3, 43]. Pre- and postnatally, there is an age-dependent maturation of the immune responses, both in cellular and humoral. Prenatal and postnatal exposure to environmental microorganisms activates the neonatal immune system and accelerates this maturation process [43].

### Toll-like receptors

Toll-like receptors (TLRs) are key elements in activation of the innate response. Several studies investigated the expression pattern of TLRs on neonatal immune cells. Levy et al. [44] and Dasari et al. [45] showed that the expression of TLRs on monocytes and granulocytes did not significantly differ between newborns and adult. In contrast, Quinello et al. [46] and Marchant et al. [47] described that the expression of TLR2 and TLR4 on monocytes and TLR4 on mature DCs was reduced among preterm and full-term newborns compared to adults. Additionally, the possibility of neonates to activate the TLR pathway seems to be reduced. Exposure to TLR ligands results in lower TNF-α, IL-6, and IL-12/23p40 production in neonates compared to adults [44, 47]. This impaired TLR activation at birth enhances neonatal vulnerability to infections. The neonatal TLR system undergoes rapid and differential development during the first month of life. Whereas the ability to produce Th1-type cytokines in response to agonists for TLR3, TLR7, and TLR9 rapidly increases to adult levels during the first month of life, TLR4-mediated responses remain impaired at least up to 1 month of age [48]. Research by Marchant et al. [47] and Sharma et al. [49] investigated the effect of CA on the ontogeny of innate cytokines production via TLR activation. Cord blood and whole blood was collected to determine cytokine production in response to TLR activation. They showed that CA did not alter the innate cytokine production. This suggests a developmental maturation of the TLR response instead of an attenuated response due to infection.

In addition to TLRs, nucleotide-binding oligomerization domain (NOD)-like receptors (NLRs) and retinoic acid-inducing gene (RIG)-like helicases (RLHs) also play an important role in pathogen recognition. No data were found on the ontogeny and function of NLR and RLH in the fetus or neonate.

Complement activity is low among newborns compared to that of adults [50]. Antimicrobial peptides and proteins are present from early gestation, and levels increase with GA and interact with TLRs [51, 52]. Mannose-binding lectin (MBL) also interacts with TLRs by upregulation of TLR2 and TLR6 signaling. MBL levels are low among (preterm) infants, which significantly increase the risk of early- and late-onset neonatal sepses [53]. These deficiencies in complement are probably one of the causes of the increased susceptibility of neonates and preterm infants to infectious agents [52]. Additionally, neutrophil levels are low among preterm and growth-restricted infants; this further enhances their vulnerability to infections [54]. Neutrophil function is also reduced: their chemotactic ability is reduced, as well as their ability to adhere to endothelial cells [55].

As in the fetus, NK cells and phagocytic cells such as monocytes, neutrophils, and DCs orchestrate neonatal innate immune responses. The phagocytic capacity of monocytes and macrophages of neonates and preterm infants are comparable to that of adults. However, their capacity to instruct the adaptive immune response is significantly reduced [56, 57]. NK cell levels of term neonates are comparable to adult levels. Their expression of inhibitory receptors is higher than among that of adults, whereas their expression of activating receptors is lower. An important function of NK cells is the production of cytokines, especially IFN-γ, which stimulates the activation of macrophages and Th1 cells. Their capacity of producing IFN-γ is significantly higher than in adults. NK cell degranulation capacity and lytic function are reduced compared to those of adult NK cells [58].

Although neonates have higher number of DCs compared with adults, their function is reduced. A study by Quinello et al. [46] showed that all newborns had lower levels of TLR4 on cDCs compared to adults. Additionally, neonatal DCs are less efficient inducers of T cell responses at birth compared to maturated DCs [59].

### Adaptive immunity

**Fig. 1:**
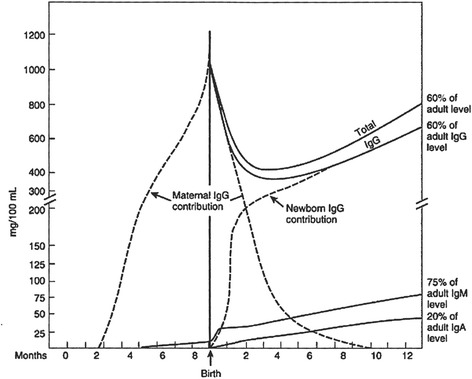
Maternal versus neonatal IgG concentrations over time (adapted from Wilson CB, Lewis DB, Penix LA. “The physiologic immunodeficiency of immaturity.” In Stiehm R, editor. *Immunologic disorders in infants and children*. 4th ed. Philadelphia: Saunders; 1996. p. 253–295)

Before birth, IgG is to some extent transmitted via the placenta between the late first trimester and 22 weeks of gestation [60]. The most important function of IgG is to facilitate innate immune mechanisms to microbial challenges the mother has already met. After 22 weeks of gestation, this transmission increases and IgG levels exceed maternal levels at birth [60]. Maternal IgG levels decline during the first weeks after birth and will increase again when the newborn itself is capable of producing its own specific IgG molecules as is displayed in Fig. 1.

A Th2-driven response characterizes both fetal and neonatal adaptive immune responses. Th1-type immune responses are downregulated in several ways. Th1 cytokine IFN-γ production is reduced [61]. Upon birth, Th2 polarization will be skewed towards a Th1 profile. Infants with delayed skewing have an increased frequency of bloodstream infections early in life [62].

Children under the age of 2 months express a combined Th2 and Th17 cell cytokine profile and display only weak Th1 polarization upon challenge with immune stimuli, which is a typical innate immune signature for newborns [42]. Given the limited exposure to antigens in early life, both T and B cells show age-dependent maturation profiles, with low numbers of class-switched memory cells detectable after birth into early infancy.

Infants born at term benefit from supplemental protection afforded by maternal antibodies transferred through the placenta. Extreme preterm infants, however, benefit to a lesser extent from transfer of maternal antibodies, which largely occurs during the third trimester of gestation [60, 63].

Adaptive immunity does play a role in fetuses and neonates but is downregulated to decrease Th1-type pro-inflammatory cytokine responses. On the one hand, this distinct functional pattern of adaptive immunity, skewed by innate immune responses, is important because Th1 cytokines, and especially TNF-α, are directly related to preterm labor and birth. On the other hand, this defensive functional expression of immunity contributes to enhanced neonatal vulnerability to infection.

## Postnatal effects of prenatal immune activation

Immunity is an interaction between all factors of defense, which are aimed to maintain host immune homeostasis. The human body is able to protect itself from infectious disease via three different strategies: avoidance, resistance, and tolerance. Avoidance reduces the risk of exposure to infectious agents. When an infectious agent invades the body, pathogen burden is reduced by resistance mechanisms, such as detection, neutralization, destruction, or expulsion of the pathogen. Tolerance strategies are aimed to reduce the negative impact of infection or inflammation on the host. The balance between the resistance and tolerance determines the degree of tissue damage in the host [64].

A substantial proportion of fetuses exposed to microorganisms in utero develop FIRS. This is associated with the production of pro-inflammatory cytokines IL-1β, IL-6, IL-8, and TNF-α, which predisposes for PPROM and premature labor. Fetuses with FIRS have a higher rate of severe neonatal morbidity. Funisitis and chorionic vasculitis, observed on pathological examination of the umbilical cord, are the histopathologic hallmarks of FIRS [65]. Funisitis is associated with endothelial activation, a key mechanism in the development of organ damage [65, 66]. The lung, gastrointestinal tract, and skin are exposed to amniotic fluid during pregnancy and are notorious targets of infection and subsequent inflammation during pregnancy. Besides the fact that antenatal infection and inflammation can predispose to organ damage, such an infection may also have an effect on organ-specific immunity. In the next part, we will summarize what is known about the effects of prenatal inflammatory exposure on skin, lung, and gut-specific immunity.

### Skin immunity

The epidermis is the first line of defense against microbial pathogens. Epidermal keratinocytes express TLRs and antimicrobial peptides belonging to the cathelicidin gene family and β-defensin family [67]. Normally, these peptides are present in low concentrations. At the time of infection, the epidermal keratinocytes start to synthesize these peptides, resulting in a rapid increase [68]. Cathelicidins have the ability to inhibit growth or destroy bacteria [69]. Cathelicidin expression is increased in the skin of both fetus and neonate, resulting in an enhanced innate immune barrier of the skin [67]. This enhanced barrier may protect the newborn from infection. CA leads to the upregulation of TLR2 and TLR4 and concomitant upregulation of cytokines and chemokines as well as antimicrobial factors in epidermal keratinocytes [70]. Neutrophils, lymphocytes, and histiocytes are the primary cells infiltrating the superficial dermis, causing dermatitis [70]. It is unknown whether this prenatal immune response of the skin influences postnatal immunity of the skin.

### Lung (RDS and BPD)

The sentinel immune cell of the lung is the alveolar macrophage [71]. No data was found on the ontogeny of alveolar macrophages in humans. Animal studies revealed that a CA infection is able to induce maturation of lung monocytes into functionally mature alveolar macrophages [72]. Human data on this topic are currently lacking.

#### Respiratory distress syndrome

RDS is a syndrome caused by lung immaturity and developmental insufficiency of surfactant production. Surfactant consists of phospholipids, neutral lipids, and proteins. There are four surfactant-associated proteins (SP), SP-A, SP-B, SP-C, and SP-D. The most important phospholipids are phosphatidylcholine (70–80%) and phosphatidylglycerol (5–10%). These phospholipids line the alveoli, in which surfactant proteins stabilize the phospholipid layer. This layer reduces the surface tension and, subsequently, neonatal work of breathing and prevents transudation of fluid. Infants with RDS have low levels of phosphatidylcholine and phosphatidylglycerol is absent, resulting in an unstable surfactant monolayer, which does not effectively reduce surface tension. Thus, infants with RDS have low lung volume, incompliant lungs, and increased work of breathing [73].

Preterm birth is associated with RDS, and it affects primarily children born before 32 weeks of gestation [74]. CA is also strongly correlated with preterm birth, but conversely, CA and intrauterine inflammation are reported to decrease the risk of developing RDS [75–78]. Histologically, CA increases the fetal pulmonary surfactant concentration in the amniotic fluid [79, 80]. It is unknown whether this CA-driven effect is pathogen-specific. Additionally, CA stimulates IL-6 production by fetal immunocompetent cells, alveolar macrophages, type II alveolar cells, and placental cells [80]. IL-6 promotes lung maturation by stimulating SP-A synthesis [80]. In turn, SP-A is able to stimulate IL-6 and TNF-α production [81, 82]. Additionally, SP-A promotes phagocytosis of specific pulmonary pathogens by alveolar macrophages [83]. An increase in SP-A might accelerate lung maturation, which decreases the incidence of RDS [79].

#### Bronchopulmonary dysplasia

Northway et al. [84] first described BPD in 1967 and defined it as a chronic lung disease affecting preterm infants with severe lung injury resulting from mechanical ventilation and oxygen toxicity. Due to the introduction of surfactant treatment for RDS, more protective ventilation strategies, and the use of antenatal glucocorticoids, BPD is now mostly seen among very immature infants, which are of a much younger GA then 30 years ago. In these infants, high concentrations of oxygen and high positive airway pressures are mostly avoided [85, 86]. In essence, these infants have a different disease compared to “old BPD.” This “new BPD” is more a clinical picture of immaturity [87]. The common denominator, however, is still stress and inflammation as a cause of histological changes and developmental delay.

Very preterm delivery is associated with CA. Many studies have examined the relationship between CA and BPD. However, the results are contradictory. A systematic review and meta-analysis by Hartling et al. [88] assessed the current available literature on the association between CA and BPD in preterm infants and showed a significant association between CA and BPD. After controlling for publication bias, results were no longer significant. Additionally, the analyses showed that GA and birth weight were confounders in the association between CA and BPD. The problem is that studies differ in their definitions used for histological CA. A study by Kim et al. [89] aimed to investigate the relationship between histological CA and neonatal morbidity using a definition of histological CA that reflects the site and extent of inflammation. Histological CA encompasses amnionitis, choriodeciduitis, and funisitis and placental inflammation. This study showed that amnionitis, which is the final stage of CA, was associated with BPD. Again, pathogen specificity was not considered. Conversely, funisitis was not associated with the development of BPD. Pro-inflammatory cytokines produced in a response to CA can be aspired by the fetus and make direct contact with respiratory epithelium, which induces pulmonary inflammation. The induction of pulmonary inflammation via the aspiration of inflammatory cytokines makes it likely that direct contact of pro-inflammatory stimuli with airway epithelium contributes to the development of BPD. This is also a possible explanation for the fact that amnionitis is associated with BPD and funisitis is not [89].

**Fig. 2:**
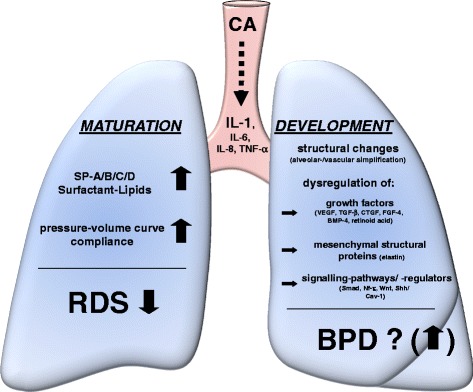
Effect of intra-amniotic inflammation/infection (IAI) on fetal lung maturation (*left side*) and development (*right side*). Kunzmann, Am J Obstet Gynecol 2013 [79]. Reprinted with permission

A study by Ambalavanan et al. [90] showed that higher levels of IL-1β, IL-8, IL-10, and IFN-γ and lower levels of IL-17, RANTES, and TNF-β were associated with the development of BPD in extremely low-birth weight infants. This cytokine pattern suggests that the outcome of BPD is associated with an impaired transition from innate to adaptive immune response mediated by T cells [90]. The association with prenatal CA, however, remains unclear. Effects of inflammation on neonatal lung development are summarized in Fig. 2.

This also leads to a relevant research question: does prenatal infection and subsequent inflammation influence the function of alveolar macrophages, the production of surfactant proteins, and local cytokine responses in the lung? Further studies are needed to address this important question.

### Gut (NEC)

The gastrointestinal (GI) tract is covered with epithelial cells, which are the first layer of defense. So-called M cells are specialized epithelium cells of the gut and are expressed in the follicle-associated epithelium of Peyer’s patches in the lower parts of the small intestine (ileum). These cells are involved in transport of antigens from the intestinal lumen to Peyer’s patches. Here, antigens are processed by antigen-presenting cells, which present the antigens to naïve T cells. Additionally, the lamina propria also contains immune cells, such as DCs and macrophages [91].

The development of the intestinal immune system starts during the fetal period. TLR2 and TLR4 are present on the fetal intestinal epithelial cell during weeks 18–21 of gestation [92]. Exposure of fetal intestinal epithelial cells to LPS/endotoxin results in higher levels of nuclear factor-κB (NF-κB) activation and IL-8 secretion compared to that of control subjects [93, 94]. IL-8 is a chemokine that recruits neutrophils. Shortly after birth, this response to endotoxin is no longer observed, indicating perinatal induction of negative regulatory control mechanisms in epithelial TLR signaling. The underlying mechanism of this hyporesponsiveness is on the one hand downregulation of IL-1-receptor-associated kinase I (IRAK1), which is essential for epithelial TLR4 signaling [94]. Downregulation of IRAK1 acts as a negative regulatory mechanism of TLR4 signaling. On the other hand, it has been long accepted that epidermal growth factor (EGF) has the potential to inhibit TLR4 signaling [95]. EGF is present in amniotic fluid [95, 96] and breast milk [97]. EGF is able to inhibit TLR4 signaling through EGF-mediated epidermal growth factor receptor (EGFR) activation [95]. These mechanisms of tolerance induction may facilitate microbial colonization in the newborn, so establishing its own microbiome. Additionally, these are important mechanisms for the induction of oral tolerance in newborns, which explains why most infants show no adverse immune reaction when exposed to environmental and dietary proteins. In this context, it is interesting that failure to downregulate TLR4 signaling and therewith an excessive IL-8 response [93] in preterm infants has been associated with the development of necrotizing enterocolitis (NEC). Since research has shown that breast milk can prevent the development of NEC, it is of great importance to develop a clinical feeding formula in which breast milk components are incorporated. Research has not yet clearly proved if EGF is the sole component in breast milk that has a protective capacity in preventing NEC. It is necessary to investigate the most effective composition of nutrition for preterm infants in order to reduce the incidence of NEC.

It was long considered that the gut is sterile at birth and that microbial colonization starts during and after delivery. The infant becomes colonized with maternal vaginal and fecal bacteria during delivery or when delivery is accomplished; via cesarean section, the infant becomes colonized with bacteria from the skin and hospital environment [98]. A review by Koleva et al. [26], however, reports preliminary evidence that microbial colonization of the gut starts in utero, which has been suggested in experimental setup by Jiménez [27]. An important research topic is the relation between prenatal gut colonization, the neonatal microbiome, and the development of NEC.

#### Necrotizing enterocolitis

NEC is one of the most common and devastating diseases among neonates with high mortality and morbidity [99, 100]. An imbalance in homeostasis leading to inflammation predisposes an infant to development of NEC. Initial bacterial colonization of the newborn gut also plays an important role in the development of NEC [93, 101]. Additionally, it seems that neonates with NEC have a different microbiome compared to neonates without NEC [101]. During the last trimester of pregnancy, the fetus swallows amniotic fluid in large quantities. Microbial DNA found in meconium suggests that the fetal intestine is already exposed to the microbes in the amniotic fluid [102]. NEC is characterized by excessive infiltration of neutrophils contributing to inflammatory necrosis [100]. Exposure of the immature or fetal enterocyte to LPS leads to an excessive production of IL-8 [93, 103]. Nanthakumar et al. [103] demonstrated that this excessive inflammatory response is caused by a developmental immaturity of innate immune response genes. Next to the developmental immaturity of the innate immune response genes, Weitkamp et al. [104] investigated that the proportion of Treg cells in the lamina propria of the intestine of premature infants is significantly reduced. The reduced amount of Treg cells may contribute to the pathology of NEC.

It is likely that antenatal inflammation is associated with the development of NEC. A systematic review and meta-analysis by Been et al. [105] investigated the association between CA and NEC. This review demonstrated that histological CA with fetal involvement was associated with a threefold increased risk of NEC and clinical CA was associated with a modest increased risk of NEC.

## Conclusions

In conclusion, we summarized the infectious and inflammatory threats for fetuses and (preterm) neonates, already present before birth. We highlighted the most prominent features of fetal and neonatal immunity and the cells and cytokines involved and also how they develop in time during gestation. We found a large number of studies focusing on prenatal infection and the host response. Maternal CA and subsequent fetal immune responses are well studied, and we found clinical data on cellular and cytokine responses to different microbial challenges. The link to postnatal immunological effects, however, turned out to be much more complicated. We found studies relating prenatal infectious and inflammatory hits to well-known neonatal diseases as well as analyses on cells and proteins involved in these processes. We also found studies with immunological-based pathogenic mechanisms for these diseases. A direct link with prenatal hits, however, could not undisputedly be established. We did however identify several unresolved topics such as neonatal consequences of labor as an inflammatory process on itself and the role of pattern recognition receptors in neonatal immunity. We also propose several questions for further research such as what is the influence of prenatal immune responses on postnatal immunity of the skin, does prenatal infection and subsequent inflammation influence the function of alveolar macrophages, the production of surfactant proteins and local cytokine responses in the lung, and what is the most appropriate nutrition for preterm infants in order to reduce the incidence of NEC? Multiple research questions on NEC remain: what is the effect of prematurity on the risk of developing NEC and how does this relate to antenatal inflammatory hits? What is the role of antenatal infection on the fetal microbiota and the host immune response and does this influence the risk of developing NEC? What cell types and cytokines are involved in NEC and what are the differences in babies with and without prenatal inflammatory hits? Further studies are needed to explore these topics.
